# An *in vitro* antiviral evaluation of punicalagin toward influenza A virus

**DOI:** 10.22038/AJP.2023.23389

**Published:** 2024

**Authors:** Fatemeh Javadi-Farsani, Ali Karimi, Hadi Razavi Nikoo, Mohammad-Taghi Moradi, Alijan Tabarraei

**Affiliations:** 1 *Student Research Committee, School of Medicine, Golestan University of Medical Sciences, Gorgan, Iran *; 2 *Cellular and Molecular Research Center, Basic Health Sciences Institute, Shahrekord University of Medical Sciences, Shahrekord, Iran*; 3 *Department of Microbiology and Virology, Golestan University of Medical Sciences, Gorgan, Iran*; 4 *Medical Plants Research Center, Basic Health Sciences Institute, Shahrekord University of Medical Sciences, Shahrekord, Iran*; 5 *Infectious Diseases Research Center, Golestan University of Medical Sciences, Gorgan, Iran*

**Keywords:** Punicalagin, Influenza, virus Antiviral agent, Mode of action

## Abstract

**Objective:**

Influenza complications are mild to serious, and can cause death in some cases. A great deal of attention has been paid in recent years to the development and use of new antiviral compounds to overcome drug resistance in certain strains of the influenza virus and treat the clinical implications. This study aimed to investigate the antiviral effect of punicalagin and its associated mechanism against influenza A (H1N1) virus *in vitro*.

**Materials and Methods:**

the ant-influenza activity of punicalagin was studied in Madin-Darby Canine Kidney (MDCK) cells using influenza virus A/Puerto Rico/8/34 (H1N1) (PR8) using Hemagglutinin assay (HA) and 50% tissue culture infective dose (TCID_50_). Then, the inhibition of haemagglutination, virucidal activity, inhibitory effect at different times, replication of viral RNA and expression of viral genes were investigated.

**Results:**

Punicalagin could inhibit influenza virus infection with 50% inhibitory concentration (IC_50_) of 3.98 μg/ml and selectivity index (SI) value of 6.1. Punicalagin decreased virus titers with an inhibitory effect on virus hemagglutination (p<0.05). Punicalagin also inhibited viral adsorption. The results of virus RNA replication and viral mRNA (NS1 and HA) expression after treatment with punicalagin showed significant suppression of viral mRNA expression but no effect on replication of viral RNA.

**Conclusion:**

The results of the present study indicated that punicalagin was effective against influenza infection most probably via inhibition of haemagglutination activity and virus binding.

## Introduction

Influenza viruses are members of the Orthomyxoviridae family with negative strand RNA segments and are divided into seven genera (Influenza A, B, C, D, infectious salmon anemia (ISA) virus, Thogoto virus and Quaranja virus (Donchet et al., 2019 ▶; Gregory et al., 2009 ▶; Palese and Shaw, 2007 ▶). Types A, B, and C influenza viruses can affect human beings. Types A and B influenza may bring about seasonal epidemics and occasionally severe complications, such as encephalitis and pneumonia. Although vaccination is recognized as an effective means of preventing the flu virus (Lee et al., 2014 ▶), it has to be updated annually due to shifts and drift in influenza surface proteins to be effective against new strains (Lee et al., 2014 ▶; Valkenburg et al., 2011). NA inhibitors, M2 ion channel inhibitors, and RNA polymerase inhibitors are used to treat influenza virus infection; the formation of influenza virus strains resistant to NA inhibitors and M2 ion channel inhibitors has recently turned out to be a serious challenge (Liu et al., 2013 ▶). Therefore, it is essential to seek out novel treatments for influenza infection. For this reason, it seems that the use of herbal compounds which inhibit virus replication is useful in the treatment of this disease. Unlimited herbal products including polyphenols, flavonoids, alkaloids, and lignans, have raised hopes as an adjunct or alternative medicine to current influenza therapies (Abdelwhab and Hafez, 2012 ▶; Guralnik et al., 2007 ▶; Kitazato et al., 2007 ▶; Moradi et al., 2017 ▶).

Punicalagin is a bioactive ellagitannin and is isolated from pomegranate (*Punica granatum*) fruit and peel. Several studies have documented the ability of various components of pomegranate fruit and its chemical constituents to inhibit a number of microorganisms (Mirjalili, 2015 ▶; Saffariet al., 2012). Pomegranate extracts have also been reported to produce effects against herpes virus, flu virus, human immunodeficiency (HIV-1) virus and poxviruses (Haidari et al., 2009 ▶; Kotwal, 2008 ▶; Moradi et al., 2020 ▶; Neurath et al., 2005 ▶; Zhang et al., 1995).

In this study, the anti-influenza virus activity of punicalagin and associated mechanisms were evaluated in MDCK cell cultures.

## Materials and Methods

**Cell and virus**

MDCK cell and influenza A virus [A/Puerto Rico/8/34 (H1N1; PR8)] were procured from the Influenza Unit, Pasteur Institute of Iran. The cells were cultivated in Dulbecco’s Modified Eagle’s Medium (DMEM; Gibco; Thermo Fisher Scientific, Inc., Waltham, MA, USA), supplemented with 100 mg/ml streptomycin, 100 U penicillin (Gibco; Thermo Fisher Scientific, Inc., Waltham, MA, USA), and 10% fetal bovine serum (FBS; Gibco; Thermo Fisher Scientific, Inc., Waltham, MA, USA), at 37°C in a 95% humidified atmosphere of 5% CO_2_.

Before infection, MDCK cells were rinsed with phosphate buffered saline (PBS) and cultivated in DMEM with pen/strep and 0.8 μg/ml L-1-tosylamide-2- phenylethyl chloromethyl ketone (TPCK) trypsin (Sigma-Aldrich, St. Louis, MO, USA).

Influenza virus was amplified in MDCK cells, and then the virus titer was measured by the TCID_50_ method and hemagglutination (HA) assay (see following sections). The virus was kept as a viral primary seed and stored at -70°C for further studies.

**Compounds**

Punicalagin was purchased from Sigma Company (Sigma-Aldrich, St. Louis, MO, USA; CAS Number:65995-63-3) and was dissolved in dimethyl sulfoxide (DMSO) and PBS to obtain a completely uniform solution. The maximum concentration of residual DMSO in cell wells was less than 0.2%, which would not have a significant effect on the experiment results.

**Cytotoxicity and antiviral activity**

The 50% cytotoxic concentration (CC_50_) of punicalagin on the MDCK cells was determined by 3-(4,5-dimethylthiazol- 2-yl)-2,5-diphenyltetrazolium bromide (MTT) method (Sigma-Aldrich, St. Louis, MO, USA) as mentioned in previous studies (Moradi et al., 2020 ▶; Mosmann, 1983 ▶).

For designation of the anti-influenza virus activity of punicalagin, MDCK cells were seeded on 12 well plates and infected with 100TCID_50_ of the virus and incubated for 1 hr at 37°C. After that the virus was removed and the infected cells were treated with 30, 15, 7.5, and 3.25 µg/ml of punicalagin and 0.8 μg/ml trypsin TPCK. After 24 and 48 hr incubation at 37°C, supernatant from infected cells was collected and the virus yield was measured by TCID_50_ method and HA assay (Kim et al., 2010 ▶). In HA assay, 50 µl two-fold serial dilutions of the culture supernatants were added to 96-well U-shape microplates and mixed with the same volume of 0.5% chicken red blood cells. The plates were kept for 45 min at room temperature and HA pattern was determined (Jang et al., 2014 ▶). For TCID_50_ virus titration, MDCK cells were seeded on 96 well plates and infected with serial 10-fold (10^-1^ – 10^-8^) dilutions of the culture supernatants in DMEM with 0.8 μg/ml trypsin TPCK and incubated at 37°C and 5% CO_2_ for 48 hr. After 48 hr incubation at 37°C, the cytopathogenic effects in each well were identified by the HA experiment and TCID_50_ values were calculated according to Reed and Muench method and expressed as TCID_50_/ml (Reed and Muench, 1938 ▶).

The antiviral effect was determined by the difference of viral titers between the treated infected cells and untreated infected cells and expressed as virus inhibition rate (IR, %). IR was calculated according to the following formula (Krylova et al., 2020 ▶):

IR = (1 − T/C) × 100%

Where T is the antilog of the compound-treated viral titers, and C is the antilog of the control (without compound) viral titers. The fifty percent inhibitory concentration (IC_50_) was calculated using a regression analysis of the dose-response curve. Selectivity index (SI) was calculated as the ratio of CC_50_ to IC_50_.

**Hemagglutination inhibition (HI) assay**

To examine the ability of punicalagin to prevent virus particles from binding to cell surface receptors, we used the hemagglutination inhibition (HI) assay as described previously (He et al., 2011 ▶). Briefly, 25 μl of two-fold nontoxic concentration of punicalagin (30 and 15 μg/ml) was made in 96-well U-shape micro-plates and mixed with 25 μl of the virus (4 HAU/50 μl). After incubation for 1 hr at room temperature, 50 μl of 0.5% chicken red blood cells was added and plates were kept for 45 min at room temperature and HA pattern was determined.

**Virucidal experiments**

Punicalagin at a maximum nontoxic concentration (30 μg/ml) and serum-free DMEM (as a control) were incubated for 1 hr at room temperature with 10^4^ TCID_50_ of the influenza virus and then the infections titer of the virus was measured by TCID_50_ method and compared with the control (Matusevich et al., 2015 ▶).

**Time-of-addition experiments**

To study the effect of punicalagin on the stage of the viral life cycle, 90% confluence cells in 6-well plates were incubated with 10^4^ TCID_50_ of the influenza virus for 1 hr at 37ºC, and then DMEM containing 0.8 μg/mL trypsin TPCK and 30 µg/ml of punicalagin was added to the cell culture before (-2 to -1 hr), simultaneously with (-1 to 0 hr), and at three time intervals after infecting (0-2, 2- 4, and 4–8 hr). After incubation at 37°C and 5% CO_2_ for 8 hr, supernatants from infected cells were separated and virus yield was measured by TCID_50_ method (Matusevich et al., 2015; Zarubaev et al., 2015).

**Preparation of cells for real-time PCR**

We used quantitative real-time PCR to evaluate the effect of the compound on viral RNA synthesis and viral mRNA expression. 80% confluence MDCK cell were infected with 100 TCID_50_ of the influenza virus in 6-well plates and incubated at 37°C for about 1 hr, and then the virus was removed and DMEM with

0.8 µg/ml of TPCK-trypsin containing 30 or 15 μg/ml of punicalagin was added (MOCK-infected sample was considered positive control). After 8 hr of incubation, cell culture supernatants were removed and total intracellular RNA was extracted from the treated and untreated cells by Trizol (Sigma-Aldrich, St. Louis, MO, USA) as per the manufacturer's guideline.

**Inhibition of viral RNA synthesis and mRNA expression**

The total RNA was transformed to cDNA by means of cDNA synthesis kit and influenza A viral RNA-specific universal primer (5’-AGCAAAAGCAGG-

3’) (Hoffmann et al., 2001) for the detection of viral RNA (vRNA) or oligo- dT primers for detection of viral mRNA (vmRNA) as per the manufacturer's guideline. The effect of punicalagin on viral RNA synthesis was calculated by absolute quantification. Six ten-fold serial dilutions of the stock virus (1.6×10^8^ TCID_50_/ml) were prepared and viral RNAs were extracted from these serial dilutions. The Ct values of the dilution range were utilized to plot a standard curve and a best- fit line was drawn. Viral RNA quantity of the samples, as log10 TCID50/ml equivalents, was obtained by plotting the Cts of the samples on the standard curve (Falsey et al., 2003 ▶). The expression levels of viral NS1 and HA mRNAs were assessed by real-time qRT-PCR using the relative quantitative method (2-ΔΔCt). GAPDH gene as the internal control was used to detect significant changes. Primers (Table 1) were designed and synthesized by Pishgaman Inc (Tehran, Iran). Real- time qRT-PCR reactions were carried out by means of SYBR Green Master Mix Kit and Rotor Gene TM 3000 (Corbett, Australia).

**Table 1:** Primers used in real-time qRT-PCR

**Gene name**	**Primer sequence**	**Product length (bp)**
*Hemagglutinin (HA)*	F:CCTGCTCGAAGACAGCCACAACG R:TTCCCAAGAGCCATCCGGCGA	98
*Nonstructural protein (NS1)*	F:CATAATGGATCCAAACACTGTGTC R:CCTCTTAGGGATTTCTGATCTCGG	138
*GAPDH*	F: GGAGAAAGCTGCCAAATATGACGA R: CGAAGGTGGAAGAGTGGGTGT	140

GAPDH: Canis lupus familiaris glyceraldehyde-3-phosphate dehydrogenase

**Statistical analysis**

The Kruskal-Wallis test was used to assess inter-group differences and p<0.05 was considered significant. The CC_50_ and IC_50_ values were calculated by using a four-parameter logistic regression. Statistical analyses were performed using GraphPad Prism 8.0 software (GraphPad Software, La Jolla, CA).

**Ethical considerations**

The study was approved by Ethics Committee of the Shahrekord University of Medical Sciences (IR.SKUMS.REC.1397.315) and Ethics Committee of the Golestan University of Medical Sciences (IR.GOUMS.REC.1397.216).

## Results

**Cytotoxicity and anti-influenza virus activity**

**Figure 1:**
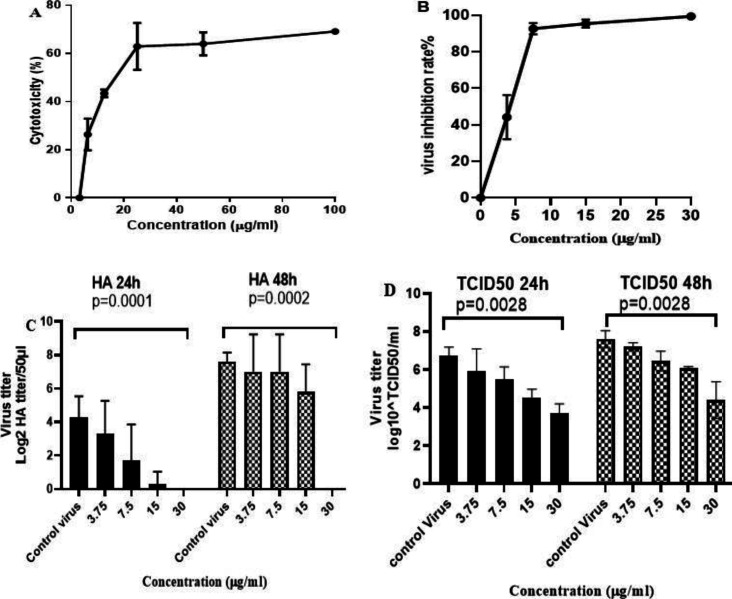
cytotoxicity and antiviral activity of punicalagin against influenza virus. A: The cytotoxicity of punicalagin was evaluated in MDCK cells using MTT assay; B: The antiviral effect was determined by the difference of viral titers between the treated infected cells and untreated infected cells and expressed as virus inhibition rate; C,D: Comparison of influenza virus titers in different concentrations of punicalagin for 24 and 48 hr and the supernatants were used for (C) Hemagglutination endpoint test and (D) 50% tissue culture infectious dose (TCID_50_) method. P value was calculated using Kruskal-Wallis nonparametric test. The data are mean±SD (standard deviation) of three independent experiments

The results showed that the CC50 value of punicalagin on MDCK cells was 24.24 μg/ml (CI95%: 16.82-34.94). The analysis showed a significant relationship between the concentration of the compound and cell death (the greater increase in the extract concentration was accompanied by higher death rate) (Figure 1A, p<0.05). MDCK cells were inoculated with PR8 virus for 1 hr. Then, the virus was removed and the cells were treated with nontoxic concentration of punicalagin. Our results showed that higher extract concentration was associated with higher virus inhibition rate (Figure 1B, p<0.05). Based on regression analysis, the IC_50_ of the punicalagin was 3.98 μg/ml (CI95%:3.68- 4.27) (Figure 1B) with SI value of 6.1. The CC_50_ and IC_50_ of oseltamivir as control drug were 539.4 (CI95%: 378.9-768.5) and 0.873 μM (CI95%: 0.55-1.37) with SI value of 617.8.

Antiviral activity of the punicalagin against influenza virus was assessed by HA endpoint test and TCID_50_ method. According to the results, punicalagin significantly decreased the levels of viral titers dose-dependently (Figure 1C, D).

**Punicalagin effects on adsorption of virus infection**

**Figure 2:**
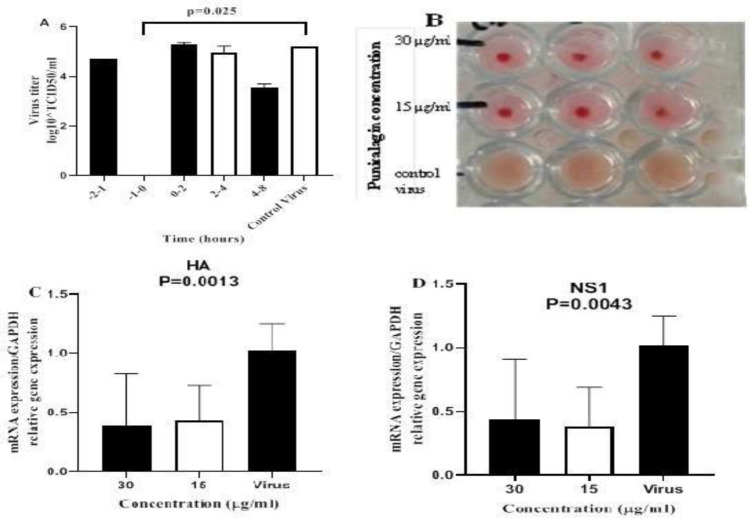
Effect of punicalagin on viral replication. A: Time-of-addition activity, the cell monolayers containing the virus were treated with the highest non-toxic concentration of punicalagin (30 µg/ml) at different times and after 8 hr (one cycle of virus replication) the virus titers were calculated in wells using TCID_50_ method. B: Haemagglutination inhibitory (HI) activity; two-fold nontoxic concentration of punicalagin (15 and 30 µg/ml) mixed with 4 haemagglutination units of the virus and the haemagglutination activity was tested by incubation with chicken RBC. C and D: Suppression of viral mRNA synthesis, the viral mRNAs for (C) HA and (D)NS1 were amplified by qRT-PCR using total RNA extracted from the MDCK cells at 8 hr after the inoculation. P values were calculated against untreated sample (virus control) using Kruskal-Wallis test

The antiviral effect of punicalagin was studied at various times of infection. The results showed that punicalagin inhibited influenza virus replication at the adsorption step (-1 to 0 hr) but did not affect the post-adsorption step of virus replication (0 to 2, 2 to 4 and 4 to 8 hr) or receptor binding step (-2 to -1 hr) (Figure 2A).

**Virucidal effect**

The virucidal activity of punicalagin against influenza virus indicated that virus titer in punicalagin and control virus groups was 5.2±0.5 and 5.3±0.28 (log10- TCID_50_), respectively (p>0.05). Therefore, the compound did not show any virucidal effect.

**Inhibition of HA**

To examine the ability of punicalagin to prevent virus particles from binding to cell surface receptors, we performed HA inhibitory method. Results showed that pretreatment with punicalagin prevented the binding of the virus to RBCs (Figure 2B).

**Viral RNA synthesis and mRNA expression**

To assess the effect of punicalagin on viral RNA synthesis and viral mRNA expression, real-time PCR was used. Results showed significant suppression of viral mRNA (NS1 and HA) expression within the cells inoculated with punicalagin treated influenza virus (p<0.01; Figure 2C). The results of virus RNA replication after treatment with punicalagin (30 and 15 µg/ml) showed that these concentrations had no effect on virus RNA replication (p>0.05, Table 2).

**Table 2:** Effect of punicalagin on viral RNA copy number

**Gene**	**Sample**		**Ct**	**Viral genes copy number (log10)**
	Virus Control		8.65±0.50	5.09±0.03
*HA*	punicalagin Concentration (µg/ml)	30	9.98±0.27	4.71±0.30
		15	8.12±0.22	5.04±0.06
	Virus Control		8.67±2.14	5.06±0.36
*NS1*	punicalagin Concentration (µg/ml)	30	9.92±1.82	4.85±0.30
	15	7.98±0.19	5.18±0.03

Cells were infected with influenza virus and treated with 30 and 15 µg/ml of punicalagin. After 8 hr of incubation, cell culture supernatants were removed and total intracellular RNA was extracted and the Log10 copy numbers of viral RNA quantity in the samples, expressed as log10 TCID_50_/ml equivalents, is derived by plotting the Ct of a sample on the standard curve, there is no significant difference between the treated and untreated samples (p>0.05, Kruskal-Wallis test).

## Discussion

Here, the effect of punicalagin on virus titer was evaluated using hemagglutination and TCID_50_ methods at 24 and 48 hr intervals after treatment. Based on our findings, punicalagin with IC_50_ value of about 3.98 μg/ml (CI95%:3.68-4.27) and SI value of 6.19, induced a substantial decline in the viral titer dose-dependently. Punicalagin is a bioactive ellagitannin and is isolated from pomegranate fruit and peel. Few studies have shown the effects of pomegranate and its chemical constituents to inhibit a number of microorganisms (Saffari et al., 2012). In our previous study, the anti-influenza virus effects of pomegranate peel ethyl alcohol extract were reported (Moradi et al., 2020 ▶). In addition, the antiviral effect of the fruit of this tree on herpes viruses has been identified (Zhang et al., 1995).

Our results regarding the effect of punicalagin on the inhibition of hemagglutination showed that punicalagin exhibited an inhibitory effect on hemagglutination of the influenza virus. In line with our study, Haidari et al. also revealed that pomegranate extract inhibited hemagglutination of influenza A virus (H1N1) (Haidari et al., 2009 ▶). In another analysis, punicalagin showed antiviral effects, which primarily acted by damaging the virus capsid or its outer envelope or penetrating the virus and destroying the virus genome (Sundararajan et al., 2010). Pomegranate juice has also been reported to inhibit HIV-1 entry (Neurath et al., 2005 ▶) which is consistent with our results. Besides that, polyphenolic compounds produce an inhibitory effect by inhibiting hemagglutination activity and changing the physical properties of the virus membrane (Song et al., 2005).

We found that punicalagin had no virucidal effect on the influenza virus, which is consistent with the Moradi et al. report. In that report, pomegranate peel extract (30 μg/ml) had no virucidal effect on the influenza virus (Moradi et al., 2020 ▶).

The results regarding virus titer at different intervals showed that this compound, along with the binding of the virus, caused a significant reduction in the virus titer, which had an inhibitory effect on its replication in the binding and penetration stages of the virus. In the study of Moradi et al. pomegranate peel extract inhibited virus replication in the stages of binding and penetration as well as the early stages of virus replication (Moradi et al., 2020 ▶). So far, some plant compounds have been found to inhibit the binding of influenza virus to host cell’s surface, including sialidase mimics, sialyl glycopolymers, and hemagglutinin inhibitors (Liu et al., 2013 ▶).

In an analysis of the effect of punicalagin on mRNA synthesis and RNA replication of the influenza virus, the results showed that treatment of virus- infected cells with punicalagin did not significantly reduce RNA replication but reduced the mRNA expression. Besides that, folic acid and pomegranate juice did not affect the virus RNA replication. It has also been reported that folic acid and pomegranate juice have potential effects on enveloped viruses to prevent and treat influenza virus, HIV, HCV, and HBV (Kotwal, 2008 ▶).

This study showed that punicalagin was effective against influenza infection most probably through inhibition of haemagglutination activity and virus binding, although it is not yet known whether this compound interacts directly with the viral hemagglutinin protein or with other viral surface components. Therefore, this compound should be further characterized to be developed as an anti-influenza virus treatment.

## Conflicts of interest

The authors have declared that there is no conflict of interest.
